# Temporal and Spatial Variation in, and Population Exposure to, Summertime Ground-Level Ozone in Beijing

**DOI:** 10.3390/ijerph15040628

**Published:** 2018-03-29

**Authors:** Hui Zhao, Youfei Zheng, Ting Li, Li Wei, Qing Guan

**Affiliations:** 1 Key Laboratory for Aerosol-Cloud-Precipitation of China Meteorological Administration, Nanjing University of Information Science and Technology, Nanjing 210044, China; zhaohui_nuist@163.com; 2 Key Laboratory of Atmospheric Environment Monitoring and Pollution Control, Collaborative Innovation Center of Atmospheric Environment and Equipment Technology, Nanjing University of Information Science and Technology, Nanjing 210044, China; c2015liting@163.com (T.L.); 20161244361@nuist.edu.cn (L.W.); 20161219415@nuist.edu.cn (Q.G.)

**Keywords:** ground-level ozone, atmospheric oxidation, exposure, China

## Abstract

Ground-level ozone pollution in Beijing has been causing concern among the public due to the risks posed to human health. This study analyzed the temporal and spatial distribution of, and investigated population exposure to, ground-level ozone. We analyzed hourly ground-level ozone data from 35 ambient air quality monitoring sites, including urban, suburban, background, and traffic monitoring sites, during the summer in Beijing from 2014 to 2017. The results showed that the four-year mean ozone concentrations for urban, suburban, background, and traffic monitoring sites were 95.1, 99.8, 95.9, and 74.2 μg/m^3^, respectively. A total of 44, 43, 45, and 43 days exceeded the Chinese National Ambient Air Quality Standards (NAAQS) threshold for ground-level ozone in 2014, 2015, 2016, and 2017, respectively. The mean ozone concentration was higher in suburban sites than in urban sites, and the traffic monitoring sites had the lowest concentration. The diurnal variation in ground-level ozone concentration at the four types of monitoring sites displayed a single-peak curve. The peak and valley values occurred at 3:00–4:00 p.m. and 7:00 a.m., respectively. Spatially, ground-level ozone concentrations decreased in gradient from the north to the south. Population exposure levels were calculated based on ground-level ozone concentrations and population data. Approximately 50.38%, 44.85%, and 48.49% of the total population of Beijing were exposed to ground-level ozone concentrations exceeding the Chinese NAAQS threshold in 2014, 2015, and 2016, respectively.

## 1. Introduction

Stratospheric ozone can protect the Earth’s surface from dangerous ultraviolet radiation from the sun, but ground-level ozone (O_3_) is an important atmospheric pollutant and agent of climate change [1]. Ground-level ozone is mainly produced by a complex photochemical reaction between nitrogen oxides (NO_X_ = NO + NO_2_) and volatile organic compounds (VOCs) under strong solar radiation. Ozone, the third most important greenhouse gas in terms of radiative forcing [2], is an important factor affecting air quality. The measured ozone concentrations are due to the combination of the hemispheric background level, intercontinental transport, stratospheric intrusion, and local and regional ozone production [3,4,5,6,7]. High ground-level ozone concentration has attracted worldwide attention from both the scientific and regulatory communities due to its adverse impacts on air quality, human health, crops, forests, materials, and climate change [8,9,10,11,12,13,14,15,16,17,18,19]. As valuable tools for ozone risk and exposure assessment, the generation of optimal ozone maps is challenging [6].

To study the spatial and temporal characteristics of ground-level ozone, a large number of long-term monitoring sites have been established worldwide, especially in the United States and Europe. For instance, Vingarzan et al. [20] reported rising ozone background levels over the midlatitudes of the Northern hemisphere at rates ranging from approximately 0.5 to 2% per year. Derwent et al. [21] reported that the trend in annual baseline ozone increase, over a 20-year period from 1987 to 2007, was 0.31 ± 0.12 ppb per year at the atmospheric research station in Mace Head, Ireland. Jaffe et al. [22] showed that O_3_ increased significantly, with a mean trend of 0.26 ppbv per year at seven out of nine sites in the Western US. Ozone precursor emission and solar radiation in the Mediterranean are usually high, especially during the summer months. A trend analysis of the European ozone monitoring site data from 1993 to 2005 showed that some Mediterranean cities recorded one-hour mean ozone concentrations exceeding 300 μg/m^3^ [23,24]. Lelieveld et al. [25] reported that summer ozone concentrations were 2.5–3 times higher in the Mediterranean area than in the background troposphere.

Given rapid economic growth and accelerated urbanization, China has been facing a complex regional air pollution challenge, characterized by high concentrations of particulate matter with an aerodynamic diameter smaller than 2.5 μm (PM_2.5_) and O_3_, especially in economically developed regions, such as Beijing-Tianjin-Hebei, the Yangtze River Delta, and the Pearl River Delta [26]. To control air pollution in China, the Chinese government introduced the Air Pollution Prevention and Control Action Plan (the “Action Plan”) in 2013. According to a study of the effects of the Action Plan’s implementation [27], the mean concentrations of PM_2.5_, particulate matter (PM_10_), sulfur dioxide (SO_2_), and oxides of nitrogen (NO_X_) showed a downward trend from 2013 to 2017. However, a reduction in PM_2.5_ and O_3_ precursors could actually lead to an increase in ground-level ozone concentration [28]. Some studies have reported that ground-level ozone has overtaken PM_2.5_ as the main summertime air pollutant in 74 Chinese cities [29]. Thus, ground-level ozone pollution in China has become a hot research topic in the atmospheric environment field and requires further research.

Meteorological factors and ozone precursors are also closely related to one another; all factors influence the concentration of ground-level ozone in the ambient air [30,31,32]. Xing et al. [5] showed that ozone precursors significantly increased in China (NO_X_: +4.3%/year; VOCs: +2.3%/year) between 1990 and 2010. Lin et al. [33] found that the probability of ozone air quality standard exceedances was strongly correlated with temperature, suggesting that an increase in temperature would aggravate ozone pollution. Zhang et al. [34] reported that summertime ground-level ozone concentrations increased during periods of high temperatures in the Northeastern United States. Tarasova et al. [35] suggested that 70% of the day-to-day ozone variability could be explained by meteorological condition changes in temperature, relative humidity, and wind speed. Wind speed and direction were also found to affect the long-range transport of ground-level ozone and the movement of its precursors from polluted areas [36].

Human health is also affected by exposure to ground-level ozone. A large number of epidemiological studies have found an association between ambient ozone levels and premature mortality. For the protection of human health, the ozone concentration standards for daily maximum 8-h and 1-h ozone concentrations set by the World Health Organization (WHO) [37] are based on associations between daily mortality rates and ozone concentrations. For class 1 (remote) areas, the National Ambient Air Quality Standards (NAAQS) of China (GB 3095-2012) established daily maximum values of 100 μg/m^3^ and 160 μg/m^3^ for 8-h and 1-h ozone concentrations, respectively. For class 2 (urban/industrial and surrounding rural) areas, these values are 160 and 200 μg/m^3^, respectively [38]. Although numerous studies have focused on health impact assessments of ground-level ozone pollution [39,40,41], these are less common in China.

Beijing has long suffered from serious ground-level ozone pollution, especially in summer [42,43,44]. Several studies on ground-level ozone have been completed in Beijing, and these studies have mostly focused on temporal and spatial variations, regional transport, production processes, and formation regimes. For example, Tang et al. [45] found that ground-level ozone concentrations in Beijing increased at a rate of 1.1 ± 0.5 ppbv/year from 2001 to 2006. Wang et al. [46] demonstrated that regional pollution sources contributed more than 34–88% to the peak ground-level ozone level at an urban site in Beijing, with an average contribution of 62%. Tang et al. [47] concluded that high amounts of ground-level ozone are strongly stimulated by non-methane volatile organic compounds (NMVOC) emissions, but the NO_X_ emissions strongly inhibit ground-level ozone formation. Notably, stringent air pollution control measures implemented by the local government during various international events have been conducive to the reduction of pollutants, except for ground-level ozone [48]. However, due to the lack of sufficient observational data, ground-level ozone concentration data from a few monitoring stations or model simulations have been used by these previous studies, considerably reducing the reliability of the results. Therefore, studying ground-level ozone pollution in Beijing using spatial interpolation using a large amount of ground-level ozone observation data that covers the entire study area is an accurate and reliable method.

In this study, hourly ground-level ozone concentration data were continuously collected at 35 sites in Beijing over the summer. The main goals of this study were to determine the temporal and spatial characteristics of ground-level ozone concentration during the summers in Beijing from 2014 to 2017 and to estimate the risk to the total population of Beijing from ground-level ozone in summer.

## 2. Methods

### 2.1. Study Area

Beijing (40° N, 116° E), the capital city of China, is located in Northern China, with 16 administrative subdivisions including six urban districts (Dongcheng, Xicheng, Zhaoyang, Fengtai, Haidian, and Shijingshan) and 10 suburban districts (Changping, Daxing, Mentougou, Tongzhou, Fangshan, Shunyi, Pinggu, Huairou, Minyun, and Yanqing). The city has a population of over 20 million and covers an area of 16,800 km^2^. Beijing has undergone rapid economic growth since the 1980s, accompanied by sustained growth in energy consumption. However, economic development has created a series of atmospheric pollution problems with especially severe ground-level ozone pollution in the summer.

### 2.2. Data

**Figure 1:**
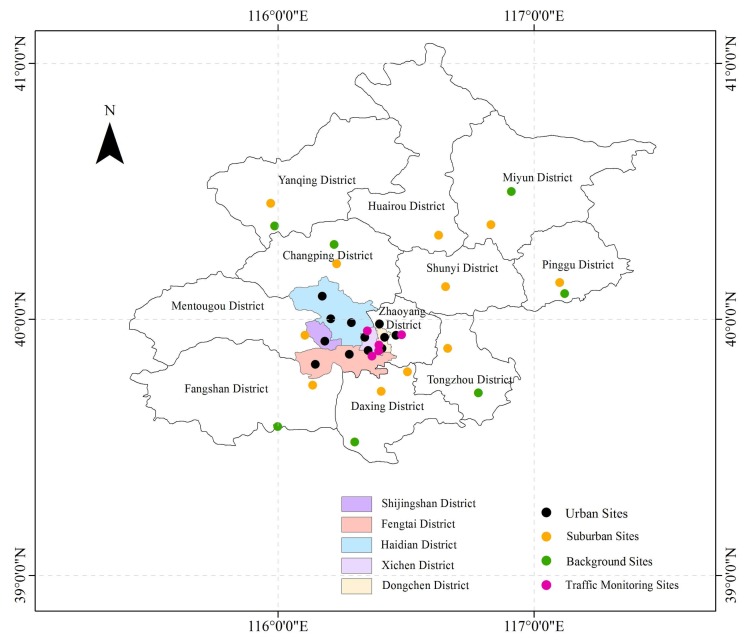
Locations of 35 ambient air quality monitoring sites in Beijing.

The Beijing Municipal Environmental Protection Bureau publishes real time monitoring concentration data for six major air pollutants on a web platform (http://zx.bjmemc.com.cn/), offering a unique resource for researchers to study the characteristics of air quality and air pollutants in Beijing. In this paper, ground-level ozone concentration data from 35 air quality monitoring sites in Beijing were recorded by the web platform. All the sites are listed in Table 1, and the locations are described in Figure 1. The sites were classified into four types according to the monitoring function [49]: 12 urban sites, 11 suburban sites, 5 traffic monitoring sites, and 7 background sites. We used the hourly ground-level ozone concentration data for the summer months, which include June, July, and August from 2014 to 2017.

Hourly meteorological data, including air temperature (*T*), relative humidity (*RH*), precipitation (*P*) and wind speed (*WD*), were collected from the China Meteorological Data Sharing Service System Administration (http://data.cma.cn/site/index.html). We obtained hourly meteorological data only for 2016 and 2017.

In addition, the population data from Beijing’s 16 districts were obtained by the Beijing statistical yearbook for every year from 2015 to 2017, as shown in Table 2.

### 2.3. Statistical Models

As a traditional statistical model, the multiple linear regression model is used to study the association between two or more independent variables and a single continuous dependent variable. The overall model can be written as:

Y = C_0_ + C_1_X_1_ + C_2_X_2_ + … C_n_X_n_

where Y is the dependent variable, X_1_, …, X_n_ are the independent variables, and C_1_, …, C_n_ are the estimated regression coefficients. In our study, Y was the ozone concentration, and C_1_, …, C_n_ were the meteorological factors (air temperature, relative humidity, and wind speed).

### 2.4. Analysis

For temporal analysis, the daily mean concentration for all of Beijing city was calculated by averaging concentrations reported by all 35 sites. Diurnal variations in ground-level ozone concentration for the four types of sites were determined by averaging the concentrations at various time points. The Chinese ground-level ozone standards (NAAQS-2012) state that the daily maximum 8-h and 1-h ozone concentrations for class 1 (remote) areas are 100 μg/m^3^ and 160 μg/m^3^, respectively, whereas for class 2 (urban/industrial and surrounding rural) areas, these values are 160 and 200 μg/m^3^, respectively.

According to the administrative divisions of Beijing, Beijing city is divided into five regions, including the center (Dongchen, Xichen, Zhaoyang, Haidian, Fengtai, and Shijingshan Districts), northwest (Yanqing and Changping Districts), northeast (Huairou, Miyun, Shunyi, and Pinggu Districts), southwest (Mentougou and Fangshan Districts), and southeast (Tongzhou and Daxing Districts). We calculated the annual mean concentrations of ground-level ozone in each region, based on the location of each air monitoring site.

The Inverse Distance Weighted (IDW) interpolate method was used to characterize spatial distribution, based on concentration data from the 35 monitoring sites. Many papers have studied the spatial distribution of air pollutants using this method [50,51,52,53]. We produced IDW-based interpolation maps of ground-level ozone concentration by randomly using 70% of the points, and the maps were validated with the remaining 30%. The Root Mean Square Error (RMSE) of each ground-level ozone concentration map was calculated to determine the accuracy of the interpolation maps using the Geostatistical Analyst Extension of ArcGIS ArcMap 10.0 (ESRI, Redlands, CA, USA).

## 3. Results

### 3.1. Variation in Ground-Level Ozone Concentration in Beijing

**Figure 2:**
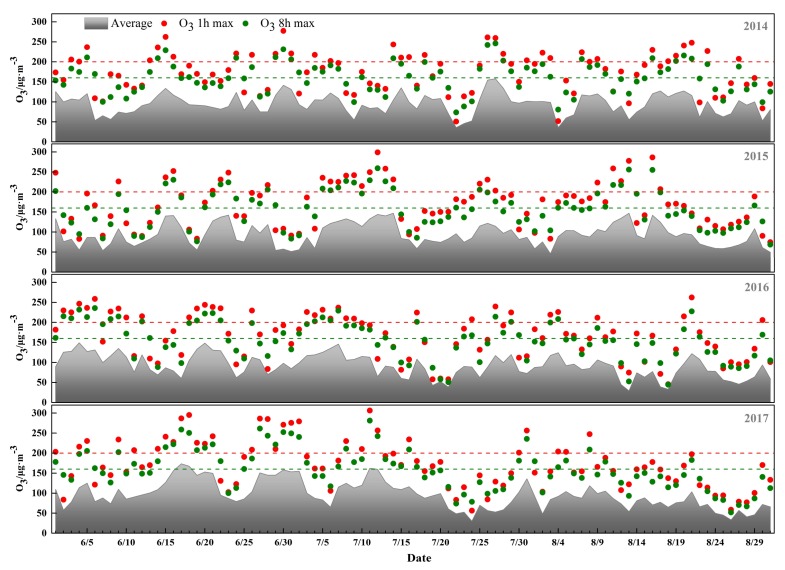
The daily mean ozone concentration and the daily maximum 1-h and 8-h ozone concentrations in different years.

Figure 2 shows the daily mean ozone concentration and the daily maximum 1-h and 8-h ozone concentrations. The daily mean ozone concentration in 2014 ranged from 35.8 to 157.6 μg/m^3^, with a mean of 94.6 μg/m^3^. From 2014 to 2016, annual mean concentrations slightly declined with fluctuations, with 91.3 μg/m^3^ being the lowest in 2016, and the daily concentration ranged from 29.4 to 149.2 μg/m^3^. In 2017, the daily mean ozone concentration ranged from 30.3 to 173.4 μg/m^3^, with a mean of 95.6 μg/m^3^, which was the highest of the four years. In addition, 44, 43, 45 and 43 days exceeded the current NAAQS threshold for ground-level ozone in 2014, 2015, 2016, and 2017, respectively.

**Figure 3:**
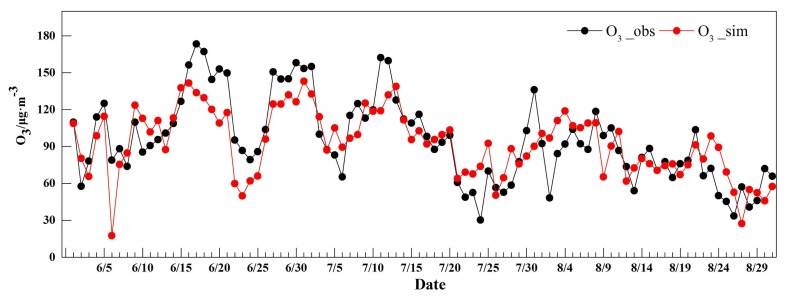
Daily variations in observed and simulated ground-level ozone concentrations.

We also compared meteorological parameters for the periods both when ground-level ozone did and did not exceed the NAAQS threshold, as shown in Table 3. The air temperature was higher during the periods when ground-level ozone exceeded the NAAQS threshold than when ground-level ozone did not exceed the NAAQS threshold. Conversely, relative humidity, wind speed, and precipitation during periods when ground-level ozone exceeded the NAAQS threshold were lower than when ground-level ozone did not exceed the NAAQS threshold. We could not further analyze the mechanism of ground-level ozone formation due to the lack of monitoring of ground-level ozone precursors and other meteorological parameters. To study the impacts of temperature, relative humidity, and wind speed on ozone levels, a multiple regression model between ground-level ozone and meteorological factors was established based on the 2016 data. The model was expressed as ozone = 6.68 × *T* − 0.79 × *RH* + 6.74 × *WS* − 3.17 with a relatively higher determinant coefficient (*R*^2^ = 0.55, *p* < 0.05). Additionally, meteorological data for 2017 were used to simulate ground-level ozone concentration to verify the predictability of the model, as shown in Figure 3. We found that the simulated results and the observed ground-level ozone concentration agreed well with each other, with a few differences.

### 3.2. Variations in Ground-Level Ozone Concentration at Four Types of Sites

**Figure 4:**
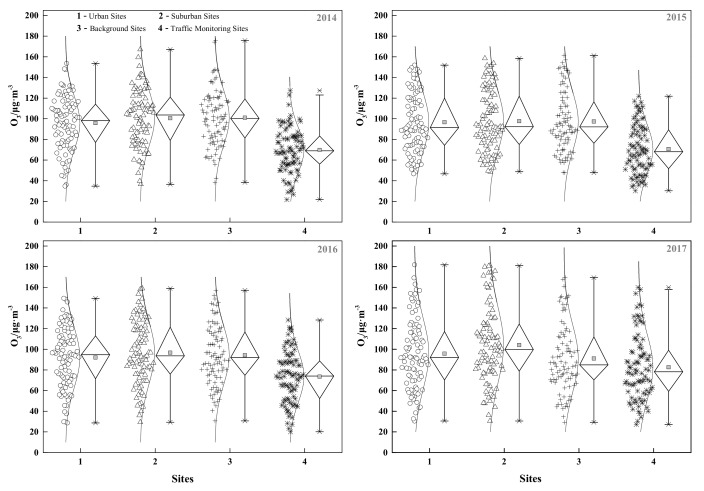
Ground-level ozone concentrations at four types of monitoring sites for different years.

As shown in Figure 4, the mean summer ozone concentrations at suburban sites in Beijing in 2014, 2015, 2016, and 2017 were 100.6, 97.7, 96.7, and 104.1 μg/m^3^, respectively, which were higher than those in urban sites. We also found that mean ozone concentrations at the traffic monitoring sites in 2014, 2015, 2016, and 2017 were 69.8, 70.6, 73.5, and 82.7 μg/m^3^, respectively, which were lower than the other three types of sites. 

**Figure 5:**
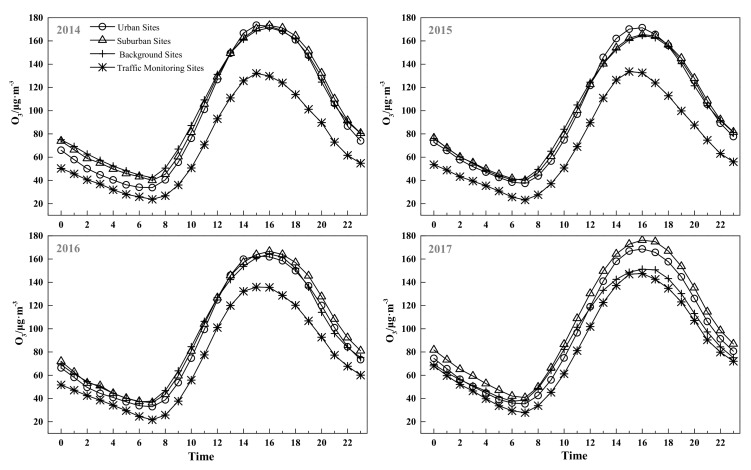
Diurnal variations of ground-level ozone concentrations at four types of monitoring sites in different years.

The diurnal variations in ground-level ozone concentration at the four types of monitoring sites are depicted in Figure 5. Overall, although the ground-level ozone concentration varied each year, the basic pattern of the curve showed that the diurnal variation in ground-level ozone at traffic monitoring sites was lower than the other three site types. The four types of monitoring site displayed similar diurnal variation, with ground-level ozone concentrations being lowest in the morning at around 7:00 a.m.—about a half hour after sunrise. The traffic monitoring stations had the lowest values in the morning, at less than 30 μg/m^3^, and the other three sites’ lowest ground-level ozone concentrations values were 30–50 μg/m^3^. Ground-level ozone concentrations increased rapidly from 7:00 a.m. until around 3:00–4:00 p.m. for all four sites, when they reached their peak. Except for 2017, the highest ground-level ozone concentrations were around 160–180 μg/m^3^ for urban, suburban, and background sites, and the maximum recorded values for the traffic monitoring sites were around 120–140 μg/m^3^. Thereafter, ground-level ozone concentration decreased steadily until the next morning. After midnight, the ground-level ozone remained low and entered a relatively stable phase.

### 3.3. Spatial Distribution of Ground-Level Ozone Concentration

We further analyzed the mean ozone concentration in each region of Beijing for different years, as shown in Table 4. We found that northeast Beijing had the highest ground-level ozone concentration of the five regions from 2014 to 2017, followed by the northwest and southeast regions. Moreover, Beijing’s center had lower ground-level ozone concentrations than the rest of the regions for 2014, 2015, and 2017.

**Figure 6:**
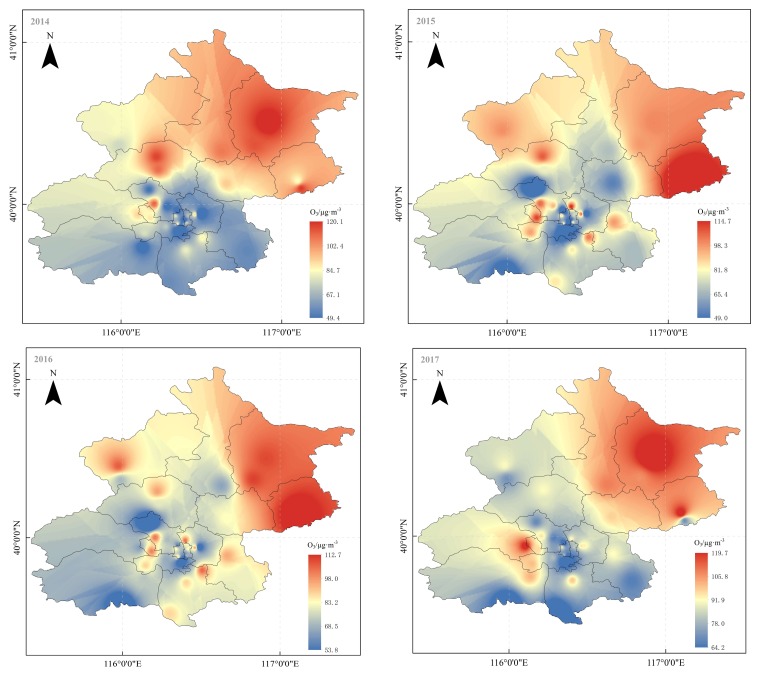
Spatial distribution of ground-level ozone concentration in Beijing in different years.

Based on inverse distance weighted interpolation and the ground-level ozone concentration data from 35 air monitoring sites, the spatial distribution of the mean ozone concentration in Beijing during the summer from 2014 to 2017 is shown in Figure 6. The RMSEs of the ground-level ozone maps in 2014, 2015, 2016, and 2017 were 7.89 (about 8.79%), 8.67 (about 10.21%), 10.03 (about 11.11%), and 7.43 μg/m^3^ (about 9.48%). Overall, ground-level ozone concentration showed a pronounced decreasing gradient from the north to the south.

### 3.4. Population Exposure to Ground-Level Ozone

**Figure 7:**
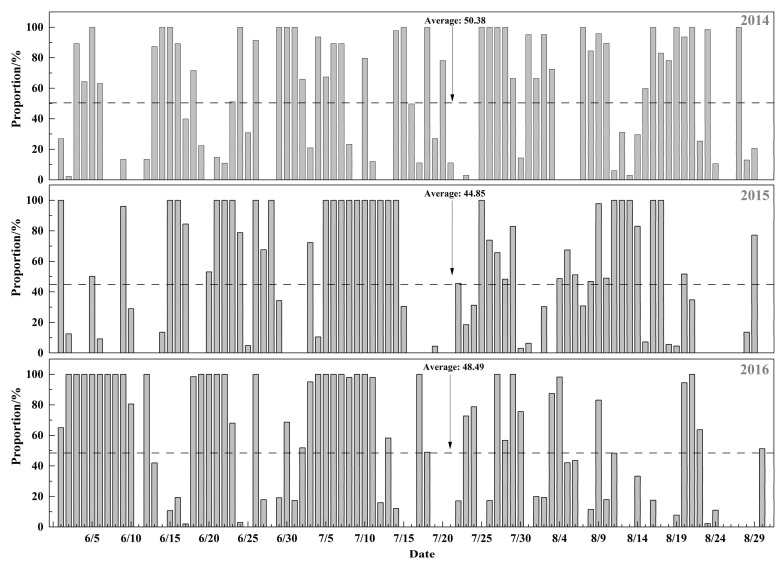
The proportion of the population exposed to ground-level ozone concentrations exceeding the National Ambient Air Quality Standards (NAAQS) of China. The daily 8-h maximum ozone (O_3_) concentration was 160 μg/m^3^ during the summer in Beijing.

Figure 7 shows the proportions of the population exposed to ground-level ozone concentrations exceeding the NAAQS of China in the summer in Beijing. Overall, about 10.84 million people (50.38%) in Beijing during the summer of 2014 were exposed to ground-level ozone concentrations exceeding the NAAQS of China, which was higher than in other years. This was due to the ground-level ozone concentration in densely populated areas, like Changping District and Haidian District, which frequently exceeded the current NAAQS threshold in 2014. In 2015, the number of people exposed to ground-level ozone concentrations exceeding the current NAAQS threshold was about 9.73 million, accounting for 44.85% of the population, which was the lowest out of the three years. A total of approximately 10.54 million, or 48.49%, of the total Beijing population was exposed to ground-level ozone concentrations exceeding the NAAQS of China in 2016.

## 4. Discussion

Rapid economic growth and urbanization in China has led to increased air pollution, especially in economically developed regions. The Chinese government has formulated and implemented many air pollution control policies and regulations to improve air quality. According to the annual national environmental situation bulletin, ground-level ozone has replaced PM_2.5_ as the main pollutant in Beijing, as of 2013. Thus, ground-level ozone pollution has become one of the top environmental issues due to its effects on ecosystems, human health and materials. Based on the hourly ground-level ozone concentration data for all 35 ambient air quality monitoring sites in Beijing during the summers of 2014 to 2017, the temporal and spatial distribution of, and population exposure to, ground-level ozone were analyzed in this paper.

In this study, we found that mean ozone concentrations at suburban sites were higher than those in urban sites. This phenomenon was essentially consistent with a few previous studies [54,55]. Ozone formation is driven by two main components of directly emitted precursors: NO_X_ and VOCs. However, ozone generation is a complex nonlinear relationship with its precursors. A few studies showed that local ozone production depends on the ratio of VOC to NO_X_ [56,57], which can explain why high ground-level ozone concentrations usually occur in the suburban areas [7]. In this case, ground-level ozone increased with increasing VOC, but decreased with increasing NO_X_. In general, low VOC to NO_X_ ratios are observed in urban areas due to the high concentration of NO_X_, and NO_X_ tends to inhibit ozone formation [6]. Therefore, higher ground-level ozone concentrations are found in suburban compared with urban areas, because ozone levels are higher downwind of ozone precursor sources at distances of hundreds or even thousands of kilometers [2,6,58]. Higher biogenic VOC emissions, low ozone titration by NO, and ozone and/or precursor transport from urban areas are factors that explain the higher ozone pollution at suburban sites [59,60]. In addition, biogenic volatile organic compounds emitted by vegetation and anthropogenic VOCs emitted by human activities are both commonly present in suburban sites [61], which can also contribute to the formation of ground-level ozone. In addition, mean ozone concentrations at the traffic monitoring sites were lower than the other three types of sites. This phenomenon can be explained by the high number of motor vehicles at the traffic monitoring sites, which produce a large amount of exhaust, leading to the increase in the emission of ozone precursors (NO and NO_2_). Although the high NO_2_ concentrations at the traffic monitoring sites are conducive to the formation of ground-level ozone through photolysis, ozone can react with NO to convert it into NO_2_ and O_2_. In addition, the NO_2_ loss processes in polluted regions are the final loss processes for ground-level ozone [62].

Increased solar radiation and temperature and decreased relative humidity can increase the rates of photochemical reactions that enhance the ozone level in the atmosphere, which explains why the ground-level ozone had an obvious diurnal variation pattern. During the daytime, with an increase in solar radiation and temperature, the atmospheric photochemical processes of ozone production also increase, which leads to the constant conversion of precursors (VOCs, NO_X_, and others) to ground-level ozone [7]. Additionally, ozone at high altitudes is transported to the ground due to atmospheric vertical diffusion during this period [20,63,64]. The ground-level ozone remained low at night because of the lack of NO_2_ photolysis reactions and photooxidation of CO, VOC_S_, and other ozone precursors. This coincides with the rapid destruction of O_3_ by NO with no replenishment from the stratosphere occurring by night due to the stable air temperature profile [6,65]. This diurnal pattern is similar to those found in several cities [31,38,66].

Spatially, ground-level ozone concentrations in Northern Beijing were higher than those in Southern Beijing. Previous studies found that the PM_2.5_ concentration in Northern Beijing was lower than in Southern Beijing [67,68], suggesting that the surface solar radiation was weaker in Southern Beijing than in Northern Beijing, which was conducive to the formation of ground-level ozone through photochemical reactions. Furthermore, Northern Beijing is surrounded by the mountains with a considerable amount of green vegetation. VOCs emitted from this green vegetation may also contribute to regional ozone formation in the Northern part of Beijing [54,61]. Conversely, Southern Beijing has more motor vehicles, resulting in increased vehicle exhaust, leading to increased NOx emission and thus strongly inhibiting ground-level ozone formation [47].

A large number of studies reported population exposure to PM_2.5_ in Beijing [67,69,70,71], and these studies were based on satellite data and model simulation results, which affects the reliability of the conclusions. However, a few studies attempted to study population exposure to ground-level ozone in Beijing. Consequently, to investigate the risk caused by summertime ground-level ozone for the entire Beijing population, the proportion of the population exposed to ground-level ozone concentrations exceeding the current NAAQS threshold was calculated by using the daily maximum 8-h ground-level ozone concentration of 160 μg/m^3^ and population survey data from all of Beijing’s 16 districts. Our research showed a higher risk of ground-level ozone exposure in Beijing. Taken together, this study reveals the significantly high health risk from ground-level ozone pollution and indicates a future direction for air pollution control. Given these results, the Chinese government must formulate environmental policies to reduce ground-level ozone pollution and the population’s exposure and achieve sustainable development. In future epidemiologic studies, the relationship between ground-level ozone and mortality, such as from chronic cardiovascular and respiratory diseases, should be established.

## 5. Conclusions

During the summer in Beijing from 2014 to 2017, more than 40 days had a ground-level ozone concentration exceeding the current NAAQS threshold. We established a multiple regression model between the meteorological parameters and ground-level ozone concentration to study the impact of air temperature, relative humidity, and wind speed on ozone levels. The test results indicated that the model could be used to simulate ground-level ozone concentration based on observational meteorological data. Of the different types of monitoring sites, the traffic monitoring sites showed the lowest ground-level ozone concentrations due to the fast titration of ozone by NO. The mean ozone concentrations at the suburban sites were higher than those at the urban sites, which could be explained by the ratio of VOC to NO_X_. In addition, the VOCs emitted from vegetation and human activities could also contribute to the formation of ground-level ozone. The diurnal variation in ground-level ozone concentration displayed a single-peak curve, with the minimum and maximum mean concentrations occurring at around 7:00 a.m. and 3:00–4:00 p.m., respectively. Overall, the ground-level ozone concentration displayed a pronounced spatial gradient, increasing from the south to the north of the region, due to the differences in vehicle exhaust, emitted VOCs, and PM_2.5_ concentration between Northern and Southern Beijing. A major challenge for assessing the health impacts of ground-level ozone pollution is the lack of monitoring data in China. Based on ground-level ozone concentrations and the population data of 16 districts in Beijing, we found that the number of individuals in the population exposed to ground-level ozone concentrations exceeding the NAAQS of China in 2014, 2015, and 2016 were 10.84 million, 9.73 million, and 10.54 million, respectively, accounting for approximately 50.38%, 44.85%, and 48.49% of the Beijing population.

High levels of ground-level ozone pollution are posing a serious threat to human health, according to our research. We propose the following recommendations to effectively reduce ground-level ozone pollution and improve the air quality in Beijing:

1. Strict laws are required to control the emissions of ozone precursors (NO_X_ and VOCs), which are mainly from motor vehicle exhaust. In the short term, replacing old cars with newer vehicles, or eliminating old cars with subsidies, are useful policy strategies. In the long term, developing public transportation to help reduce the use of private cars in major and populous cities is also required.
2. To avoid an increase in ground-level ozone concentrations, measures for controlling VOC sources (like gasoline stations and paint use) should be stricter than those for NO_X_ sources, because most areas in Beijing are VOC-dominated ozone pollution areas, where the ozone concentration is determined by the concentration of VOC in the atmosphere. Thus, the relevant research community and government agencies need to pay more attention to research on the measurement of VOCs and their effects on ozone production in the atmosphere.
3. As previous studies have shown, ozone pollution in Beijing is considerably affected by the transport of ozone and its precursors from other provinces in Northern China. Regional cooperation and joint defense and control are future directions for the control of ozone pollution.
4. Research on the impacts of ground-level ozone pollution on human health and vegetation is limited compared to that on atmospheric processes. We recommend that more research be conducted on the impacts of ground-level ozone and consideration be given to further reducing the guideline values for Class II regions.

## Figures and Tables

**Table 1:** Types and locations of air quality monitoring sites in Beijing.

Site ID	Type	Location
Dongsi	Urban Sites	116.42° E, 39.93° N
Temple of Heaven		116.41° E, 39.89° N
West Park Officials		116.34° E, 39.93° N
West Wanshou Nishinomiya		116.35° E, 39.88° N
Olympic Sports Center		116.40° E, 39.98° N
Agricultural Exhibition Hall		116.46° E, 39.94° N
Wanliu		116.29° E, 39.99° N
Northern New Area		116.17° E, 40.09° N
Botanical Garden		116.21° E, 40.00° N
Fengtai garden		116.28° E, 39.86° N
Yungang		116.15° E, 39.82° N
Shijingshan city		116.18° E, 39.91° N
Liangxiang	Suburban Sites	116.14° E, 39.74° N
Daxing		116.40° E, 39.72° N
Yizhuang		116.51° E, 39.80° N
Tongzhou		116.66° E, 39.89° N
Shunyi		116.66° E, 40.13° N
Changping		116.23° E, 40.22° N
Mentougou		116.11° E, 39.94° N
Pinggu		117.10° E, 40.14° N
Huairou		116.63° E, 40.33° N
Miyun		116.83° E, 40.37° N
Yanqing		115.97° E, 40.45° N
Dingling	Background Sites	116.22° E, 40.29° N
Badaling		115.99° E, 40.37° N
Miyun Reservoir		116.91° E, 40.50° N
Donggaocun		117.12° E, 40.10° N
Yongledian		116.78° E, 39.71° N
Yufa		116.30° E, 39.52° N
Liulihe		116.00° E, 39.58° N
Qianmen East Street	Traffic Monitoring Sites	116.40° E, 39.90° N
Yongdingmen Inner Street		116.39° E, 39.88° N
Xizhimen North Street		116.35° E, 39.95° N
South 3rd Ring Road		116.37° E, 39.86° N
East 4th Ring Road		116.48° E, 39.94° N

**Table 2:** The population for Beijing’s 16 districts from 2014 to 2016.

District	2014 Population (Thousands)	2015 Population (Thousands)	2016 Population (Thousands)
Fengtai	2300	2324	2255
Fangshan	1036	1046	1096
Tongzhou	1356	1378	1428
Dongchen	911	905	878
Zhaoyang	3922	3955	3856
Haidian	3678	3694	3593
Daxing	1545	1562	1694
Xichen	1302	1288	1259
Yanqing	316	314	327
Shijingshan	650	652	634
Mentougou	306	308	311
Shunyi	1004	1020	1075
Pinggu	423	423	437
Huairou	381	384	393
Changping	1908	1963	2010
Miyun	478	479	483

**Table 3:** Comparison of meteorological parameters during different periods.

	Air Temperature (°C)	Relative Humidity (%)	Wind Speed (m/s)	Precipitation (mm)
Ozone does not exceed the National Ambient Air Quality Standards (NAAQS) threshold	24.82	73.85	1.81	0.32
Ozone exceed the NAAQS threshold	26.87	64.10	1.63	0.11

**Table 4:** Ozone mean concentration in each region of Beijing in different years.

Region	O_3_ (μg/m^3^)			
	2014	2015	2016	2017
Center	88.2 ± 16.6	88.8 ± 7.4	86.6 ± 14.9	91.9 ± 10.4
Northwest	105.5 ± 9.8	99.8 ± 4.0	97.5 ± 8.0	98.9 ± 19.7
Northeast	110.7 ± 7.9	100.5 ± 10.6	101.9 ± 10.6	107.0 ± 11.3
Southwest	90.1 ± 12.7	89.0 ± 17.0	81.1 ± 8.8	92.0 ± 10.0
Southeast	91.3 ± 7.3	97.5 ± 4.5	95.6 ± 4.3	96.0 ± 4.8
